# 1-(3,5-Dichloro­phen­yl)-3-(2-meth­oxy­phen­yl)triaz-1-ene

**DOI:** 10.1107/S1600536812005132

**Published:** 2012-02-17

**Authors:** Mohammad Reza Melardi, Maryam Aghamohamadi, Jafar Attar Gharamaleki, Mohammad Kazem Rofouei, Behrouz Notash

**Affiliations:** a Department of Chemistry, Islamic Azad University, Karaj Branch, Karaj, Iran; b Faculty of Chemistry, Tarbiat Moallem University, Tehran, Iran; c Department of Chemistry, Shahid Beheshti University, G.C., Evin, Tehran 1983963113, Iran

## Abstract

The title mol­ecule, C_13_H_11_Cl_2_N_3_O, is almost planar and adopts a *trans* conformation with respect to the –N=N– bond; the dihedral angle between the rings is 3.47 (2)°. The N—N bond lengths indicate the presence of single- and double-bond characters and hence the –N=N—NH– moiety. In the crystal, inversion dimers linked by pairs of N—H⋯Cl hydrogen bonds occur, and C—H⋯π and π–π stacking interactions are also observed.

## Related literature

For background literature and the synthesis of related compounds, see: Rofouei *et al.* (2009 ▶). For the synthesis and mol­ecular structure of a similar monochloro-substituted triazene, see: Rofouei *et al.* (2012 ▶).
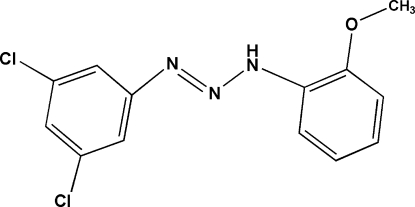


## Experimental

### 

#### Crystal data

C_13_H_11_Cl_2_N_3_O
*M* *_r_* = 296.15
Monoclinic, 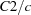

*a* = 15.422 (3) Å
*b* = 23.068 (5) Å
*c* = 7.6141 (15) Å
β = 92.60 (3)°
*V* = 2706.0 (9) Å^3^
*Z* = 8
Mo *K*α radiation
μ = 0.47 mm^−1^
*T* = 298 K
0.5 × 0.3 × 0.15 mm

#### Data collection

Stoe IPDS 2T diffractometer
15133 measured reflections
3659 independent reflections
2178 reflections with *I* > 2σ(*I*)
*R*_int_ = 0.162

#### Refinement

*R*[*F* ^2^ > 2σ(*F* ^2^)] = 0.066
*wR*(*F* ^2^) = 0.137
*S* = 1.07
3659 reflections
177 parameters
1 restraint
H atoms treated by a mixture of independent and constrained refinement
Δρ_max_ = 0.24 e Å^−3^
Δρ_min_ = −0.28 e Å^−3^

### 

Data collection: *X-AREA* (Stoe & Cie, 2005 ▶); cell refinement: *X-AREA*; data reduction: *X-RED32* (Stoe & Cie, 2005 ▶); program(s) used to solve structure: *SHELXS97* (Sheldrick, 2008 ▶); program(s) used to refine structure: *SHELXL97* (Sheldrick, 2008 ▶); molecular graphics: *ORTEP-3 for Windows* (Farrugia, 1997 ▶); software used to prepare material for publication: *WinGX* (Farrugia, 1999 ▶).

## Supplementary Material

Crystal structure: contains datablock(s) I, global. DOI: 10.1107/S1600536812005132/pv2511sup1.cif

Structure factors: contains datablock(s) I. DOI: 10.1107/S1600536812005132/pv2511Isup2.hkl

Supplementary material file. DOI: 10.1107/S1600536812005132/pv2511Isup3.cml

Additional supplementary materials:  crystallographic information; 3D view; checkCIF report

## Figures and Tables

**Table 1:** Hydrogen-bond geometry (Å, °) *Cg*1 is the centroid of the C2–C7 ring.

*D*—H⋯*A*	*D*—H	H⋯*A*	*D*⋯*A*	*D*—H⋯*A*
N1—H1⋯Cl1^i^	0.85 (2)	2.69 (2)	3.529 (2)	170 (3)
C1—H1*C*⋯*Cg*1^ii^	0.96	2.76	3.553 (4)	140

Symmetry codes: (i) 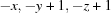; (ii) 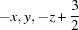.

**Fig. 1.**
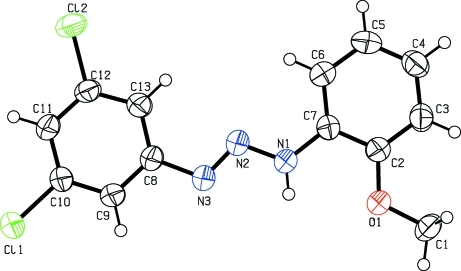
Molecular structure of the title compound with ellipsoids drawn at 50% probability level.

**Fig. 2.**
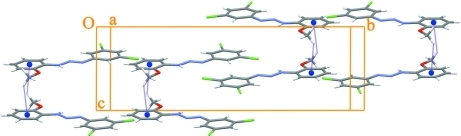
Part of the unit-cell packing of the title compound showing C—H···π interactions between a methoxy H atom and the phenyl ring with a H···π distance of 2.76 Å.

## supplementary crystallographic information

### Comment

In continuation of our studies on the synthesis and characterization of trizene compounds as ligands in our laboratory (Rofouei *et al*., 2012; Rofouei *et al.*, 2009), we now report the crystal structure of the title compound.

The title molecule (Fig. 1) adopts *trans* configuration about the (–N2═N3–) bond and is almost planar with the dihedral angel between two aromatic rings 3.47 (2) °. Non-Classic N—H···Cl hydrogen bond with D···A distance of 3.529 (2) Å connect the individual molecules into dimers. The N1—N2 and N2—N3 bond distances are 1.323 (3) and 1.256 (3) Å, which indicate the presence of a single and a double bond characters, respectively. Another interesting feature of the title compound is the presence of π–π [*Cg*1···*Cg*1 distance of 3.757 (2) Å] and edge-to-face C1—H1C···*Cg*1 stacking interactions between the methoxy hydrogen and the phenyl ring with H···π distance of 2.76 Å, in which *Cg*1 is the center of (C2—C7) ring. Unit cell packing diagram of the title compound is presented in Fig. 2, showing N—H···Cl hydrogen bonds.

The bond distances and bond angles in the title compoiund are in agreement with the corresponding bond distances and bond angles reported for a closely related structure, [1-(2-methoxyphenyl)-3-(4-chlorophenyl)]triazene (Rofouei *et al.*, 2012).

### Experimental

To a 1 L flask in an ice bath, was added dichloroaniline (6.36 g, 0.05 mol) and HCl (4.68 g, 0.13 mol; d = 1.18 g.ml^-1^). To the obtained solution was added dropwise a solution of sodium nitrite (4.14 g in 25 ml H_2_O). Then, a diluted solution of *o*-anisidine (6.15 g, 0.05 mol) in methanol (10 ml) was added to the solution. The pH of the solution was adjusted at about 7–8 by adding a solution of sodium acetate ( 14.76 g, 0.18 mol) in 45 ml H_2_O as solvent. The solution was stirred for about 45 minutes, giving an orange precipitate. It was then filtered off and dried under vacuum. After dissolving in dichloromethane and recrystallization, orange crystals of the title compound were obtained.

### Refinement

N—H hydrogen atom were found in a difference Fourier map and refined isotropically with distance restraint of 0.85 (2) Å. All C—H hydrogen atoms were positioned geometrically and refined as riding atoms with C—H = 0.93 and 0.96 Å, *U*_iso_(H) = 1.2*U*_eq_(C) and 1.5*U*_eq_(C) for aryl and methyl H atoms, respectively.

### Crystal data

**Table:**

C_13_H_11_Cl_2_N_3_O	*F*(000) = 1216
*M**_r_* = 296.15	*D*_x_ = 1.454 Mg m^−^^3^
Monoclinic, *C*2/*c*	Mo *K*α radiation, λ = 0.71073 Å
Hall symbol: -C 2yc	Cell parameters from 3659 reflections
*a* = 15.422 (3) Å	θ = 1.6–29.4°
*b* = 23.068 (5) Å	µ = 0.47 mm^−^^1^
*c* = 7.6141 (15) Å	*T* = 298 K
β = 92.60 (3)°	Needle, orange
*V* = 2706.0 (9) Å^3^	0.5 × 0.3 × 0.15 mm
*Z* = 8	

### Data collection

**Table:**

Stoe IPDS 2T diffractometer	2178 reflections with *I* > 2σ(*I*)
Radiation source: fine-focus sealed tube	*R*_int_ = 0.162
Graphite monochromator	θ_max_ = 29.4°, θ_min_ = 1.6°
Detector resolution: 0.15 pixels mm^-1^	*h* = −21→20
rotation method scans	*k* = −31→31
15133 measured reflections	*l* = −10→10
3659 independent reflections	

### Refinement

**Table:**

Refinement on *F*^2^	Primary atom site location: structure-invariant direct methods
Least-squares matrix: full	Secondary atom site location: difference Fourier map
*R*[*F*^2^ > 2σ(*F*^2^)] = 0.066	Hydrogen site location: inferred from neighbouring sites
*wR*(*F*^2^) = 0.137	H atoms treated by a mixture of independent and constrained refinement
*S* = 1.07	*w* = 1/[σ^2^(*F*_o_^2^) + (0.0234*P*)^2^ + 2.1152*P*] where *P* = (*F*_o_^2^ + 2*F*_c_^2^)/3
3659 reflections	(Δ/σ)_max_ < 0.001
177 parameters	Δρ_max_ = 0.24 e Å^−^^3^
1 restraint	Δρ_min_ = −0.28 e Å^−^^3^

### Special details

**Table:**

Experimental. ^1^H-NMR (300 MHz, *d*^6^-DMSO) δ, p.p.m.: 3.83 (3*H*, CH_3_), 6.73–7.69 (7*H*, aromatic groups) and 12.93(1*H*, NH group). ^13^C-NMR (100 MHz, DMSO) δ, p.p.m.: 55.8 (O—CH_3_), 111.9–153.8 (C atoms of aromatic rings). IR (KBr): 3314, 1601, 1566, 1473, 1255, 754 cm^-1^.
Geometry. All e.s.d.'s (except the e.s.d. in the dihedral angle between two l.s. planes) are estimated using the full covariance matrix. The cell e.s.d.'s are taken into account individually in the estimation of e.s.d.'s in distances, angles and torsion angles; correlations between e.s.d.'s in cell parameters are only used when they are defined by crystal symmetry. An approximate (isotropic) treatment of cell e.s.d.'s is used for estimating e.s.d.'s involving l.s. planes.
Refinement. Refinement of *F*^2^ against ALL reflections. The weighted *R*-factor *wR* and goodness of fit *S* are based on *F*^2^, conventional *R*-factors *R* are based on *F*, with *F* set to zero for negative *F*^2^. The threshold expression of *F*^2^ > σ(*F*^2^) is used only for calculating *R*-factors(gt) *etc*. and is not relevant to the choice of reflections for refinement. *R*-factors based on *F*^2^ are statistically about twice as large as those based on *F*, and *R*- factors based on ALL data will be even larger.

### Fractional atomic coordinates and isotropic or equivalent isotropic displacement parameters (Å^2^)

**Table:**

	*x*	*y*	*z*	*U*_iso_*/*U*_eq_	
Cl1	0.15277 (6)	0.62694 (3)	0.38482 (14)	0.0726 (3)	
Cl2	0.38395 (5)	0.46656 (4)	0.20924 (14)	0.0697 (3)	
O1	−0.07732 (12)	0.25239 (8)	0.5479 (3)	0.0541 (5)	
N1	0.04554 (15)	0.32351 (9)	0.4565 (4)	0.0510 (6)	
N2	0.10429 (14)	0.36171 (9)	0.4104 (3)	0.0461 (6)	
N3	0.07934 (15)	0.41328 (9)	0.4243 (3)	0.0515 (6)	
C1	−0.14585 (18)	0.21619 (13)	0.6009 (5)	0.0577 (8)	
H1A	−0.1640	0.1912	0.5054	0.087*	
H1B	−0.1939	0.2397	0.6335	0.087*	
H1C	−0.1260	0.1932	0.6998	0.087*	
C2	−0.00257 (17)	0.22621 (11)	0.5001 (4)	0.0438 (6)	
C3	0.01140 (19)	0.16686 (11)	0.4955 (4)	0.0494 (7)	
H3	−0.0323	0.1414	0.5256	0.059*	
C4	0.0906 (2)	0.14548 (12)	0.4459 (4)	0.0552 (7)	
H4	0.1001	0.1057	0.4434	0.066*	
C5	0.15531 (19)	0.18293 (13)	0.4004 (4)	0.0544 (7)	
H5	0.2086	0.1684	0.3685	0.065*	
C6	0.14148 (18)	0.24231 (12)	0.4020 (4)	0.0488 (7)	
H6	0.1852	0.2674	0.3703	0.059*	
C7	0.06270 (17)	0.26414 (10)	0.4508 (4)	0.0416 (6)	
C8	0.14307 (17)	0.45444 (11)	0.3765 (4)	0.0435 (6)	
C9	0.12052 (17)	0.51217 (11)	0.3953 (4)	0.0484 (7)	
H9	0.0659	0.5222	0.4327	0.058*	
C10	0.17985 (18)	0.55468 (11)	0.3581 (4)	0.0495 (7)	
C11	0.26112 (18)	0.54131 (12)	0.3013 (4)	0.0520 (7)	
H11	0.3009	0.5703	0.2776	0.062*	
C12	0.28154 (17)	0.48354 (12)	0.2808 (4)	0.0491 (7)	
C13	0.22487 (17)	0.43961 (11)	0.3177 (4)	0.0482 (7)	
H13	0.2405	0.4010	0.3041	0.058*	
H1	−0.0038 (13)	0.3373 (12)	0.481 (4)	0.059 (9)*	

### Atomic displacement parameters (Å^2^)

**Table:**

	*U*^11^	*U*^22^	*U*^33^	*U*^12^	*U*^13^	*U*^23^
Cl1	0.0684 (5)	0.0384 (3)	0.1140 (8)	0.0020 (3)	0.0377 (5)	0.0057 (4)
Cl2	0.0474 (4)	0.0709 (5)	0.0930 (7)	0.0123 (4)	0.0281 (4)	0.0165 (4)
O1	0.0418 (10)	0.0424 (9)	0.0791 (15)	−0.0020 (8)	0.0160 (10)	0.0022 (9)
N1	0.0397 (12)	0.0396 (11)	0.0748 (18)	−0.0021 (10)	0.0150 (12)	0.0018 (11)
N2	0.0435 (12)	0.0408 (11)	0.0546 (15)	−0.0040 (9)	0.0080 (11)	0.0038 (10)
N3	0.0459 (12)	0.0395 (11)	0.0699 (17)	−0.0025 (10)	0.0140 (11)	0.0055 (11)
C1	0.0398 (14)	0.0620 (17)	0.072 (2)	−0.0061 (13)	0.0109 (15)	0.0102 (16)
C2	0.0407 (13)	0.0432 (12)	0.0476 (16)	−0.0006 (11)	0.0034 (12)	−0.0013 (12)
C3	0.0539 (16)	0.0411 (13)	0.0535 (18)	−0.0062 (12)	0.0065 (14)	0.0026 (12)
C4	0.0650 (18)	0.0421 (13)	0.059 (2)	0.0071 (13)	0.0111 (16)	−0.0018 (13)
C5	0.0501 (15)	0.0554 (15)	0.0585 (19)	0.0131 (14)	0.0107 (14)	−0.0068 (14)
C6	0.0419 (14)	0.0493 (14)	0.0559 (19)	−0.0012 (12)	0.0089 (13)	−0.0008 (13)
C7	0.0430 (14)	0.0371 (12)	0.0446 (16)	−0.0022 (11)	0.0028 (12)	−0.0007 (11)
C8	0.0427 (13)	0.0417 (12)	0.0465 (16)	−0.0021 (11)	0.0087 (12)	0.0062 (11)
C9	0.0438 (14)	0.0438 (13)	0.0589 (19)	0.0034 (11)	0.0155 (13)	0.0044 (13)
C10	0.0498 (15)	0.0369 (12)	0.063 (2)	0.0025 (11)	0.0147 (14)	0.0079 (12)
C11	0.0457 (15)	0.0467 (14)	0.065 (2)	−0.0014 (12)	0.0157 (14)	0.0126 (13)
C12	0.0399 (13)	0.0515 (14)	0.0570 (19)	0.0058 (12)	0.0151 (13)	0.0093 (13)
C13	0.0500 (16)	0.0418 (13)	0.0538 (19)	0.0057 (12)	0.0118 (14)	0.0057 (12)

### Geometric parameters (Å, º)

**Table:**

Cl1—C10	1.733 (3)	C4—C5	1.376 (4)
Cl2—C12	1.739 (3)	C4—H4	0.9300
O1—C2	1.365 (3)	C5—C6	1.386 (4)
O1—C1	1.420 (3)	C5—H5	0.9300
N1—N2	1.323 (3)	C6—C7	1.381 (4)
N1—C7	1.396 (3)	C6—H6	0.9300
N1—H1	0.854 (17)	C8—C9	1.386 (4)
N2—N3	1.256 (3)	C8—C13	1.400 (4)
N3—C8	1.426 (3)	C9—C10	1.379 (4)
C1—H1A	0.9600	C9—H9	0.9300
C1—H1B	0.9600	C10—C11	1.379 (4)
C1—H1C	0.9600	C11—C12	1.380 (4)
C2—C3	1.387 (4)	C11—H11	0.9300
C2—C7	1.398 (4)	C12—C13	1.375 (4)
C3—C4	1.386 (4)	C13—H13	0.9300
C3—H3	0.9300		
			
C2—O1—C1	117.7 (2)	C7—C6—C5	120.0 (3)
N2—N1—C7	120.8 (2)	C7—C6—H6	120.0
N2—N1—H1	116 (2)	C5—C6—H6	120.0
C7—N1—H1	123 (2)	C6—C7—N1	122.4 (2)
N3—N2—N1	113.1 (2)	C6—C7—C2	119.8 (2)
N2—N3—C8	113.0 (2)	N1—C7—C2	117.8 (2)
O1—C1—H1A	109.5	C9—C8—C13	120.1 (2)
O1—C1—H1B	109.5	C9—C8—N3	115.7 (2)
H1A—C1—H1B	109.5	C13—C8—N3	124.1 (2)
O1—C1—H1C	109.5	C10—C9—C8	119.4 (2)
H1A—C1—H1C	109.5	C10—C9—H9	120.3
H1B—C1—H1C	109.5	C8—C9—H9	120.3
O1—C2—C3	125.3 (2)	C11—C10—C9	121.7 (2)
O1—C2—C7	115.0 (2)	C11—C10—Cl1	118.6 (2)
C3—C2—C7	119.8 (3)	C9—C10—Cl1	119.6 (2)
C4—C3—C2	119.9 (3)	C10—C11—C12	117.8 (2)
C4—C3—H3	120.1	C10—C11—H11	121.1
C2—C3—H3	120.1	C12—C11—H11	121.1
C5—C4—C3	120.2 (3)	C13—C12—C11	122.5 (2)
C5—C4—H4	119.9	C13—C12—Cl2	119.5 (2)
C3—C4—H4	119.9	C11—C12—Cl2	117.9 (2)
C4—C5—C6	120.3 (3)	C12—C13—C8	118.4 (2)
C4—C5—H5	119.8	C12—C13—H13	120.8
C6—C5—H5	119.8	C8—C13—H13	120.8
			
C7—N1—N2—N3	−179.4 (3)	C3—C2—C7—N1	179.6 (3)
N1—N2—N3—C8	179.4 (3)	N2—N3—C8—C9	−177.7 (3)
C1—O1—C2—C3	1.4 (4)	N2—N3—C8—C13	0.7 (4)
C1—O1—C2—C7	−179.4 (3)	C13—C8—C9—C10	−0.9 (5)
O1—C2—C3—C4	−179.4 (3)	N3—C8—C9—C10	177.6 (3)
C7—C2—C3—C4	1.5 (5)	C8—C9—C10—C11	0.5 (5)
C2—C3—C4—C5	−0.3 (5)	C8—C9—C10—Cl1	−178.9 (2)
C3—C4—C5—C6	−0.7 (5)	C9—C10—C11—C12	0.6 (5)
C4—C5—C6—C7	0.5 (5)	Cl1—C10—C11—C12	180.0 (3)
C5—C6—C7—N1	179.3 (3)	C10—C11—C12—C13	−1.2 (5)
C5—C6—C7—C2	0.6 (5)	C10—C11—C12—Cl2	−180.0 (2)
N2—N1—C7—C6	1.8 (4)	C11—C12—C13—C8	0.8 (5)
N2—N1—C7—C2	−179.5 (3)	Cl2—C12—C13—C8	179.5 (2)
O1—C2—C7—C6	179.2 (3)	C9—C8—C13—C12	0.3 (4)
C3—C2—C7—C6	−1.6 (5)	N3—C8—C13—C12	−178.0 (3)
O1—C2—C7—N1	0.4 (4)		

### Hydrogen-bond geometry (Å, º)

*Cg*1 is the centroid of the C2–C7 ring.

**Table:**

*D*—H···*A*	*D*—H	H···*A*	*D*···*A*	*D*—H···*A*
N1—H1···Cl1^i^	0.85 (2)	2.69 (2)	3.529 (2)	170 (3)
N1—H1···O1	0.85 (2)	2.33 (3)	2.624 (3)	100 (2)
C1—H1*C*···*Cg*1^ii^	0.96	2.76	3.553 (4)	140

Symmetry codes: (i) −*x*, −*y*+1, −*z*+1; (ii) −*x*, *y*, −*z*+3/2.
